# Pragmatic clinic-based investigation of echocardiogram parameters in asymptomatic patients with type 2 diabetes in routine clinical practice and its association with suggestive coronary artery disease: a pilot study

**DOI:** 10.1186/s13098-023-01128-4

**Published:** 2023-07-22

**Authors:** Catia Cristina Silva Sousa Vergara Palma, Pablo Moura Lopes, Alfredo de Souza Bomfim, Marilia Brito Gomes

**Affiliations:** 1 Department of Internal Medicine, Diabetes Unit, State University Hospital of Rio de Janeiro, Hospital Universitário Pedro Ernesto, Av. 28 de Setembro, No. 77, 3 Andar, Vila Isabel, Rio de Janeiro, 20551-030 Brazil; 2 Department of Internal Medicine, Cardiology Unit, State University Hospital of Rio de Janeiro, Hospital Universitário Pedro Ernesto, Av. 28 de Setembro, No. 77, 2 Andar, Vila Isabel, Rio de Janeiro, Brazil

**Keywords:** Coronary artery disease, T2DM patients without cardiovascular symptoms, Echocardiogram, Framingham risk score

## Abstract

**Background:**

Patients with diabetes mellitus (DM) have cardiovascular diseases (CVD) as a major cause of mortality and morbidity. The primary purpose of this study was to assess the echocardiographic parameters that showed alterations in patients with type 2 diabetes mellitus(T2DM) with suggestive coronary artery disease (CAD) determined by electrocardiography and the secondary was to assess the relationship of these alterations with established cardiovascular risk factors.

**Methods:**

This cross-sectional, observational pilot study included 152 consecutive patients with T2DM who attended a tertiary DM outpatient care center. All patients underwent clinical examination and history, anthropometric measurements, demographic survey, determination of the Framingham global risk score, laboratory evaluation, basal electrocardiogram, echocardiogram, and measurement of carotid intima-media thickness (CIMT).

**Results:**

From the overall sample, 134 (88.1%) patients underwent an electrocardiogram. They were divided into two groups: patients with electrocardiograms suggestive of CAD (n = 11 [8.2%]) and those with normal or non-ischemic alterations on electrocardiogram (n = 123 [91.79%]). In the hierarchical multivariable logistic model examining all selected independent factors that entered into the model, sex, high triglycerides levels, and presence of diabetic retinopathy were associated with CAD in the final model. No echocardiographic parameters were significant in multivariate analysis. The level of serum triglycerides (threshold) related to an increased risk of CAD was ≥ 184.5 mg/dl (AUC = 0.70, 95% IC [0.51–0.890]; p = 0.026.

**Conclusion:**

Our pilot study demonstrated that no echocardiogram parameters could predict or determine CAD. The combination of CIMT and Framingham risk score is ideal to determine risk factors in asymptomatic patients with T2DM. Patients with diabetic retinopathy and hypertriglyceridemia need further investigation for CAD. Further prospective studies with larger sample sizes are needed to confirm our results.

## Background

Patients with diabetes mellitus (DM) have cardiovascular diseases (CVD) as a major cause of mortality and morbidity. These includes arteriosclerotic diseases (coronary heart disease, cerebrovascular disease, peripheral arterial disease) and heart failure (HF) [1]. Despite recent improvements in the treatment of DM-related chronic complications, patients with type 2 diabetes mellitus (T2DM) continue to die due to cardiovascular diseases [2]. Screening for ischemic cardiovascular disease in patients with asymptomatic DM is not routinely indicated. Even though some studies have shown that may be already silent ischemia in one in five and severe ischemia in one in 15 cases of asymptomatic patients with DM [3]. The proportion of myocardial ischemia varies from 22 to 44% among studies, and only few studies of silent myocardial ischemia (SMI) assess the incidence of the disease in this group of patients [4].

Screening for SMI in asymptomatic patients with T2DM is not well established, especially in those with intensive therapy addressed for comorbidities such as obesity, smoking, hypertension and dyslipidemia [5], despite having worse prognosis. Furthermore, an increased risk of cardiovascular death in patients with T2DM has been recently related to any degree of diabetic retinopathy (DR) [6].

Regarding examinations, the Brazilian Diabetes Association and American Diabetes Association [1, 7] recommend performing only baseline electrocardiography (ECG) annually for patients with DM. Baseline electrocardiography is a simple and inexpensive test for routine investigation of CVD in DM. Although other tests such as: ergometric test, echocardiography with pharmacological stress, coronary angio-tomography, ankle-brachial index (ABI) and the coronary artery calcium score (CAC) can assess the positivity of myocardial ischemia and subclinical atherosclerosis, they can help stratify cardiovascular risk with traditional risk factors in asymptomatic patients. Whether the use of these more expensive tests alters the treatment and prognosis of asymptomatic patients remains unclear [1, 7].

According to actual guidelines [8], echocardiogram can be done only in patients with suggestive heart disease. This method of investigation can detect structural and functional cardiac alterations such as: Left ventricular (LV) hypertrophy, decreased LV diastolic and systolic function, diastolic dysfunction (DD) (25–75%) and reduced ejection fraction (1%). It is noteworthy that some of the above mentioned alterations can also occur in other clinical conditions such as: hypertension, dyslipidemia and obesity that are also present in patients with T2DM.This results in a controversial issue about DM being an independent risk factor for the presence of those echocardiographic alterations. Considering heart disease as one of the most important cause of mortality in patients with T2DM, early identification of patients with increased risk can modify the clinical course of this disease even in patients without cardiac symptoms [9–11].

The primary purpose of the study was to assess the echocardiographic parameters that showed alterations in patients with T2DM with suggestive coronary artery disease (CAD) determined by electrocardiography and the secondary was to assess the relationship of these alterations with established cardiovascular risk factors.

## Methods

### Study design, ethics and population

This was an observational pilot, cross-sectional, study of 152 consecutive patients with T2DM who were attended at a tertiary DM outpatient care center at Policlínica Piquet Carneiro, State University of Rio de Janeiro (PPC/UERJ). The Institutional Research Ethics Committee approved the study protocol (number CAAE: 31940114.8.0000.5259). The inclusion and exclusion criteria, clinical variables, comorbidities, renal function, DR, Framingham risk score (FRS) and determination of carotid intima media thickness (CIMT) were all previously described [12].

A basal electrocardiogram (Cardiocare 2000 Bionet^®^) was performed in an appropriated environment after a resting period of 15 min by an experient cardiologist who reported the results and was blinded to the patient data, except for the diagnosis of T2DM. The final interpretation was divided into three classifications: normal reports, reports suggestive of CAD (spiked or inverted T wave, ST-segment elevation or depression, Q wave) [13] and reports of altered non-CAD. Each category was defined in accordance with the guidelines of the Brazilian Society of Cardiology [13].

Echocardiographic images were obtained by a single experienced cardiologist, in accordance with the recommendations of the American Society of Echocardiography [14]. The examinations were carried out using a Philips Eco-Color Doppler ultrasound device, model iE33, with an S5-1 (1–5 MHz) sector heart probe and L11-3 Hz linear probe (3–11 MHZ) and semi-automatic edge detection program QLAB7 0.1 during the morning, after a patient rest of at least 5 min, in a climate-controlled room with dim light. The LV ejection fraction was determined using Teichholtz method. The ejection fraction classification was as follows: reduced (< 40%), mid-range (between 40 and 49%) and preserved (≥ 50%) [9]. Vascular age (VA) was defined according to the ARIC study normality tables [15]. VA was determined by the mean CIMT value for each side (right and left) that represented the 50th percentile. This calculated VA was used instead of chronological age (CA) for cardiovascular risk stratification using the FRS.

### Statistical analysis

Firstly, we conducted an exploratory analysis, and data are presented as mean (standard deviation), median values (interquartile range, IQR) for continuous variables, and numbers (relative frequencies) for discrete variables. For comparison of variables with abnormal distribution we have used the Mann–Whitney test. For comparison between categorical variables, we have used X^2^ and Fisher tests. To further explore the relationship among CAD and demographic, laboratory, echocardiographic data, presence of DM-related chronic complications, and other traditional risk factors related to CAD, a multivariable hierarchical logistic regression (Backward Wald model) was performed to determine which variables could be associated with the presence of CAD as a dependent variable. To select the independent variables, we chose those with statistical significance in exploratory (p ≤ 0.1) or clinical plausibility. Subsequently, the order of entry into the model started with demographic and social data (gender, self-reported color-race, smoking, self- reported frequency of physical activity) followed by clinical data (myocardial infarction, abdominal circumference) and use of drugs (use of statins), laboratory data (total cholesterol, uric acid, and albuminuria), and data related to DM-related chronic complications (presence of DR) and cardiovascular evaluation, neuropathy, FRS, and parameters of echocardiography. Model fit was assessed using the Hosmer–Lemeshow and Omnibus tests. The calculated Nagelkerke R2 and the odds ratio (OR) with a 95% confidence interval (CI) were expressed where indicated. Receiver operating characteristic curve (ROC) analysis was applied to identify the level of serum triglycerides related to an increased risk of CAD with the estimated area under the ROC curve (AUC) and 95% confidence with the optimal Youden index. Statistical significance was defined as a two-sided P-value of < 0.05. SPSS version 25.0 was used for statistical analysis.

## Results

### Description of demographic and laboratory data

A total of 152 asymptomatic patients with T2DM were enrolled and an overview of the study population is presented in Table 1.

**Table 1:** Clinical and laboratory characteristics of the studied population

Data	N = 152
Demographic data	
Females, n (%)	91 (59.9)
Age (years)	54.79 ± 10.09
Age at diagnosis (years)	42.36 ± 10.67
Known diabetes duration (years)	12.64 ± 8.43
Years of school attendance	9.43 ± 4.36
Self-reported color-race (white/non-white), n (%)	39.5/60.5
Positive family history (FH)	
FH of type 2 diabetes, n(%)	113 (74.3)
FH of obesity, n (%)	65 (42.8)
FH of hypertension, n (%)	117 (77.0)
FH of premature CAD, n (%)	59 (38.8)
FH of known thyroid dysfunction, n (%)	15 (9.9)
Anthropometric data	
BMI (kg/m^2^)	30.5 ± 5.1
SBP (mmHg)	132.2 ± 15.5
DBP (mmHg)	78.9 ± 9.7
HR (bpm)	80.7 ± 13.1
Waist (cm)	99.1 ± 11.2
WHR (cm)	0.97 ± 0.09
Classic cardiovascular risk factors	
Hypertension, n (%)	114 (75.0)
Dyslipidemia, n (%)	131 (86.2)
Current smokers, n (%)	13 (8.6)
Sedentary lifestyle, n (%)	108 (71.1)
Current alcohol consumption, n (%)	62 (40.8)
Treatment data	
Basal insulin, n (%)	84 (55.6)
Metformin, n (%)	143 (94.1)
Statins, n (%)	118 (77.6)
Antihypertensive drugs, n (%)	114 (75.0)
Aspirin, n (%)	53 (35.8)
Diabetes-related chronic complications	
Retinopathy, yes, n (%)	51 (34)
Diabetic renal disease (GFR < 60 ml/min), n (%)	22 (14.3)
Neuropathy (self-reported or symptoms), n (%)	75 (48.7)
Laboratory data	
Fasting glucose (mg/dl)	172.3 ± 86.3
HbA1c (%)	8.1 ± 1.7
Total cholesterol (mg/dl)	193.3 ± 61.2
Triglycerides(mg/dl)	161 (41–820)
HDLc (mg/dl)	57.9 ± 17.9
Non_HDL (mg/dl)	135.4 ± 54.6
LDLc (mg/dl)	95.7 ± 41.2
TSH (µUI/ml)	2.1 ± 1.3
FreeT4 (ng/dl)	1.2 ± 0.1
CRP (mg/dl)	0.51 (0.01–4.29)
Uric acid (mg/dl)	6.1 ± 2.2
Albumin (g/dl)	4.7 ± 0.9
Albuminuria (mg/g)	9.5 (0.22–374.06)
GFR (ml/min/1.73 m^2^)	85.6 ± 25.1
CIMT data	
IMT on the right (mm)	0.66 ± 0.14
IMT on the left (mm)	0.70 ± 0.14
IMT on worst side (mm)	0.70 ± 0.14
Vascular age using right CIMT (years)	54.1 ± 13.5
Vascular age using left CIMT (years)	54.8 ± 13.9
Vascular age using worst CIMT (years)	58.7 ± 14.1
Framingham risk score	
Global FRS	19.1 ± 13.3
FRS with VA on worst side	22.3 ± 17.8
Echocardiographic parameters	
Aortic diameter (mm)	32.0 ± 3.7
Left atrial diameter (mm)	35.1 ± 3.3
Right ventricular diameter (mm)	15.9 ± 0.5
Left ventricular end-diastolic diameter (mm)	49.2 ± 4.7
Left ventricular end-systolic diameter (mm)	28.9 ± 3.9
Interventricular septal thickness (mm)	8.7 ± 1.2
Left ventricular mass (g)	152.8 ± 38.9
EF (%)	71.8 ± 6.1
Left ventricular end-systolic volume	33.0 ± 11.4
Left ventricular end-diastolic volume	115.7 ± 25.3
Left atrial diameter fraction-shortening (%)	58.5 ± 5.1
End-diastolic thickness of left ventricular posterior wall (mm)	9.3 ± 7.4

Data are presented as mean ± SD or median and interquartile range (IQR)

*FH* family history, *CAD* coronary artery disease, *BMI* body mass index, *SBP* systolic blood pressure, *DBP* diastolic blood pressure, *HR* heart rate, *WHR* waist to hip ratio, *GFR* glomerular filtration rate, *HDLc* high density cholesterol, *LDLc* low density cholesterol, *CRP* C protein, *CIMT* carotid intima-media thickness, *IMT* intima-media thickness, *FRS* Framingham risk score, *VA* vascular age, *EF* ejection fraction

### Description of the study population by basal electrocardiogram

**Fig. 1:**
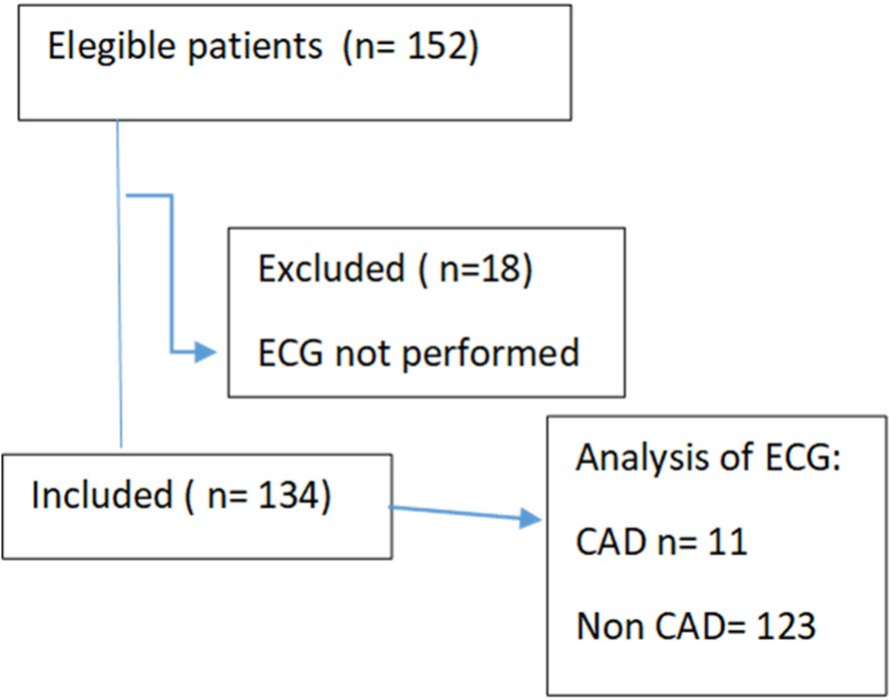
Flowchart of patients

In the overall sample, 134 (88.1%) patients performed the electrocardiogram. They were divided into two groups: patients with electrocardiograms suggestive of coronary artery disease (n = 11) and those with normal or non-ischemic alterations on electrocardiogram (n = 123). The flowchart of the selection of study subjects is presented in Fig. 1.

Differences between the two groups regarding demographic data, positive family history of T2DM, obesity, hypertension, premature CAD, anthropometric data, classic cardiovascular risk factors, treatment data, DM-related chronic complications, and laboratory data are presented in Table 2. The level of serum triglycerides (theresold) related to an increased risk of CAD was ≥ 184.5 mg/dl (AUC = 0.70, 95% IC [0.51–0.890]; p = 0.026.

**Table 2:** Clinical, treatment and laboratory differences in patients with and without coronary artery disease determined by electrocardiogram

Demographic data	N = 11	N = 123	p
Males, n (%)	8 (72.7)	46 (37.4)	0.022
Age (years)	57.55 ± 8.99	55.54 ± 9.65	0.448
Age at diagnosis (years)	42.55 ± 8.89	43.15 ± 10.71	0.780
Known duration of diabetes (years)	12.64 ± 8.43	12.65 ± 8.38	0.318
Years of school attendance	9.73 ± 3.74	9.54 ± 4.58	0.594
Self-reported color-race (white/non-white), n (%)	54.5/45.5	39/71	0.315
Positive family history (FH)			
FH of type 2 diabetes, n (%)	10 (90.9)	86 (69.9)	0.139
FH of obesity, n (%)	5 (45.5)	56 (45.5)	0.996
FH of hypertension, n (%)	9 (81.8)	91 (74.0)	0.567
FH of premature CAD, n (%)	3 (27.3)	48 (39.0)	0.442
Anthropometric data			
BMI (kg/m^2^)	30.77 ± 6.24	30.54 ± 5.11	0.932
SBP (mmHg)	137.18 ± 17.56	132.25 ± 16.00	0.260
DBP (mmHg)	82.27 ± 11.02	78.73 ± 9.61	0.290
Waist (cm)	99.18 ± 15.60	99.60 ± 1.05	0.955
WHR (cm)	0.98 ± 0.13	0.97 ± 0.09	0.894
Classic cardiovascular risk factors			
Hypertension, n (%)	9 (81.8)	93(75.6)	0.644
Dyslipidemia, n (%)	11 (100)	106 (86.2)	0.187
Current smokers, n (%)	1 (9.1)	9 (7.3)	0.830
Sedentary lifestyle, n (%)	6 (54.5)	90 (73.2)	0.189
Current alcohol consumption, n (%)	4 (36.4)	52 (42.3)	0.703
Treatment data			
Basal insulin, n (%)	8 (72.7)	66 (53.7)	0.234
Metformin, n (%)	11 (100)	115 (93.5)	0.414
Statins, n (%)	11 (100)	95 (77.2)	0.075
Antihypertensive drugs, n (%)	9 (81.8)	93 (75.6)	0.644
Aspirin, n (%)	5 (45.5)	42 (34.1)	0.502
Diabetes-related chronic complications			
Retinopathy, yes, n (%)	7 (63.6)	36 (29.3)	0.022
Diabetic renal Disease (GFR < 60 ml/min), n (%)	3 (27.27)	17 (13.8)	0.247
Neuropathy (self-reported of symptoms), n (%)	5 (81.8)	55 (45.8)	0.385
Laboratory data			
Fasting glucose (mg/dl)	186 (129)	142 (101)	0.134
HbA1c (%)	8.78 ± 1.83	7.96 ± 1.67	0.146
Total cholesterol (mg/dl)	225.73 ± 72.23	187.44 ± 54.81	0.109
Triglycerides (mg/dl)	228 (469)	160 (116)	0.026
HDLc (mg/dl)	58.79 ± 1.41	59.31 ± 1.87	0.920
Non-HDLc (mg/dl)	168.9 ± 73.46	133.96 ± 54.49	0.182
LDLc (mg/dl)	105.00 ± 49.75	95.55 ± 4.22	0.383
Uric acid (mg/dl)	7.98 ± 3.30	6.15 ± 2.11	0.061
Albumin (g/dl)	5.28 ± 1.17	4.75 ± 0.99	0.112
Albuminuria (mg/g)	26.81 ± 2.19	37.32 ± 6.48	0.071

Data are presented as mean ± SD or median and interquartile range(IQR)

*FH* family history, *CAD* coronary artery disease, *BMI* body mass index, *SBP* systolic blood pressure, *DBP* diastolic blood pressure, *HR* heart rate, *WHR* waist to hip ratio, *GFR* glomerular filtration rate, *HDLc* high density cholesterol, *LDLc* low density cholesterol, *CRP* C protein, *CIMT* carotid intima-media thickness, *IMT* intima-media thickness, *FRS* Framingham risk score, *VA* vascular age, *EF* ejection fraction

Cardiovascular assessment was performed using global FRS risk stratification, echocardiography, CIMT measurement, VA derived from IMT, and replacement of VA in FRS. The two groups were compared in terms of these parameters, and the data are presented in Table 3.

**Table 3:** Cardiovascular evaluation of patients with and without coronary artery disease determined by electrocardiogram

CIMT data	N = 11	N = 123	p
IMT on the right (mm)	0.77 ± 0.20	0.66 ± 0.13	0.054
IMT on the left (mm)	0.74 ± 0.19	0.65 ± 0.12	0.149
IMT on worst side (mm)	0.80 ± 0.20	0.69 ± 0.13	0.110
Vascular age using right CIMT (years)	61.21 ± 22.02	54.62 ± 1.35	0.082
Vascular age using left CIMT (years)	62.18 ± 15.18	54.21 ± 1.22	0.263
Vascular age using worst CIMT (years)	66.47 ± 17.34	58.60 ± 1.33	0.149
Framingham risk score			
FRS global	23.32 (20.62)	14.52 (15.07)	0.037
FRS with VA on worst side	32.64 (52.59)	16.11 (17.73)	0.018
Echocardiographic parameters			
Aortic diameter (mm)	32.18 ± 5.19	32.07 ± 3.59	0.542
Left atrial diameter (mm)	36.09 ± 3.72	35.01 ± 3.29	0.113
Right ventricular diameter (mm)	36.09 ± 3.72	16.02 ± 0.54	0.284
Left ventricular end-diastolic diameter (mm)	50.73 ± 4.22	49.10 ± 4.85	0.273
Left ventricular end-systolic diameter (mm)	28.18 ± 4.02	28.88 ± 4.04	0.691
Interventricular septal thickness (mm)	9.09 ± 1.13	8.80 ± 1.17	0.386
Left ventricular mass (g)	169.17 ± 46.69	153.25 ± 38.68	0.30
EF (%)	75.20 ± 5.64	71.78 ± 6.17	0.1
Left ventricular end-systolic volume	30.94 ± 10.63	32.93 ± 11.91	0.680
Left ventricular end-diastolic volume	123.35 ± 22.81	115.47 ± 24.77	0.262
Left atrial diameter fraction-shortening (%)	55.46 ± 5.32	58.61 ± 4.99	0.08
End-diastolic thickness of LV posterior wall (mm)	9.09 ± 1.13	9.54 ± 8.30	0.398

Data are presented as mean ± SD or median and interquartile range(IQR)

*CIMT* carotid intima-media thickness, *IMT* intima-media thickness, *FRS* Framingham risk score, *VA* vascular age, *EF* ejection fraction, *LV* left ventricular

### Description of multivariable analysis with the presence of suggestive coronary artery disease as a dependent variable

In the hierarchical multivariable logistic model examining all selected independent factors that entered into the model, sex, high triglyceride levels, and presence of DR were associated with CAD in the final model. No association was found regarding the other variables. All the independent variables which entered in the model could explain 35.3% (Nagelkerke R-squared) of a patient in our sample having suggestive CAD. When sex was replaced by the FRS with CA, no significance was found. No echocardiographic parameters were significant in the multivariate analysis. In this model, all the independent variables which entered in the model could explain 25.1% (Nagelkerke R-squared) of a given patient presenting suggestive CAD. When sex was replaced by the FRS with VA, this variable was significant. In this model, all the independent variables which entered in the model could explain 35.4% (Nagelkerke R-squared) of a patient in our sample having suggestive CAD. All data are described in Table 4.

**Table 4:** Final adjusted hierarchical multivariable logistic models: A model with gender as independent variable. B: model with FRS and VA as independent variable. C: model with FRS and CA as independent variable

Variable	B	OR	95%CI	p
A: Model with male gender as independent variable				
Triglycerides	0.006	1.006	1.002–1.010	0.003
Retinopathy (yes)	2.038	7.674	1.591–37.005	0.011
Gender, male	1.978	7.228	1.382–37.798	0.019
B: Model with Framingham risk score with VA as independent variable				
Triglycerides	0.005	1.005	1.001–1.010	0.008
Retinopathy, yes	1.993	7.340	1.494–36.062	0.014
FRS with VA on worst side	1.978	7.228	1.382–37.798	0.019
C: Model with Framingham risk score with CA as independent variable				
Triglycerides	0.006	1.006	1.002–1.010	0.002
Retinopathy, yes	1.568	4.796	1.161–19.812	0.030

A: Variable(s) entered in Step 1: total cholesterol, triglycerides, uric acid, retinopathy, albuminuria, and sex

B: Variable(s) entered in step 1: total cholesterol, triglycerides, uric acid, retinopathy, albuminuria, and FRS with VA on the worst side

C: Variable(s) entered in Step 1: total cholesterol, triglycerides, uric acid, retinopathy, albuminuria, and FRS with CA

## Discussion

Our pilot study demonstrated that no echocardiogram parameters could predict and/or determine suggestive CAD. The combination of CIMT and FRS was most effective in identifying risk factors in asymptomatic patients with T2DM.

The Brazilian Diabetes Guidelines and the American Association of Diabetes (ADA) recommend performing an annual baseline electrocardiogram in patients for determining risk scores and a better stratification of cardiovascular risk in patients with DM [1, 7]. The use of noninvasive tools associated with the optimization of clinical treatment has already been shown to be superior to several invasive procedures, according to previous studies. Referência???

In our study, males were more frequently found to present electrocardiograms suggestive of CAD than females. In a systematic review of more than 4.5 million patients with T2DM, it was observed a prevalence of CVD of 32.2% with higher rates among in men than women. In this latter study, CAD was observed in (21.2%) and stroke in (7.6%) of the patients [16]. However other studies have demonstrated a 25–50% increased risk of CVD in women with T2DM [17]. It is supposed that this occurs since women have higher blood pressure, higher frequency of endothelial dysfunction, more abnormalities in their mechanisms of fibrinolysis and thrombosis compared to those who do not develop DM [2].On the other hand, VA assumes that the shift of CA to VA derived from vascular imaging data (where CIMT may suggest the presence of subclinical atherosclerosis) could lead to refinement of adequate cardiovascular risk? [18, 19]. In symptomatic and asymptomatic patients with T2DM, previous studies [20] demonstrated that the combination of the FRS with CIMT provided superior discriminating power for cardiovascular events compared to the use of the FRS alone.

Higher levels of triglycerides were observed in the group with suggestive CAD by electrocardiogram and although 100% of the patients in this group were using statins, the majority of them were outside the therapeutic target recommended for stratified cardiovascular risk. The present study showed a level of triglycerides ≥ 184.5 mg/dl as threshold for the presence of CAD. Hypertriglyceridemia can be associated with higher circulating concentration of atherogenic remnant cholesterol or even higher atherogenic small, dense low-density lipoproteins (LDLc) and an increased risk of CVD [21]. Patients with high triglycerides levels could represent a subgroup that requires more intensive therapy since they are exposed to higher cardiovascular risk. Non- HDLc is recommended as a secondary lipid target since it is more intrinsically related to atherosclerotic CVD compared to LDLc alone. It includes not only LDLc but other proatherogenic lipoproteins such as: apolipoprotein B, lipoproteins rich in triglycerides ([TRLs]), intermediate and very low-density lipoproteins, chylomicrons and their remnants and lipoprotein(a) that are responsible for the persistent residual risk even after control of LDLc levels [22].

The presence of DR, a chronic microvascular DM-related complication, is an important factor associated with an electrocardiogram suggestive of CAD. This fact has already been evaluated in patients with DM, including those with type 1 diabetes [6, 23]. Hyperglycemia can cause small vessel damage such as diabetic macular edema (DME) and proliferative DR that are retinal microvascular hyperglycemia-induced complications. Microvascular retinal impairment (DME or PDR) may reflect a more generalized vascular impairment in patients with DM2 and, therefore, could be considered a marker of vascular impairment in other organs such as brain and heart. Although the presence of DR was a significant variable in our study, this finding should be evaluated with caution since the confidence interval was wide due to the small sample of patients.

There is a non-significant difference in A1c% of almost 1% between the groups with and without CAD determined by ECG; however, it is relevant since larger studies with more than 5,000 participants such as the UKPDS study, have demonstrated that this difference can decrease the risk of microvascular complications in patients with T2DM [24]. Other studies also confirmed a 15% myocardial infarction risk reduction for a 0.88% lower A1c% [25].

Regarding echocardiogram, the guidelines recommend that it can be performed in patients with suggestive heart disease. Many structural and functional changes can be detected by echocardiography in patients with T2DM: LV hypertrophy, decreased diastolic and systolic LV function, diastolic dysfunction (DD) (25–75%) and reduced ejection fraction (1%) [11, 26]. However, despite LV hypertrophy, increased LV mass, and DD being present in patients with T2DM, there are controversies about DM itself being an independent risk factor, since factors such as arterial hypertension, obesity, and dyslipidemia often coexist in these patients, as demonstrated in the present study [27, 28]. Early identification of subclinical LV dysfunction can identify patients at an increased risk earlier in the disease course, even in asymptomatic T2DM patients with a normal ejection fraction, allowing potential preventive therapeutic objectives [29]. Finally, as demonstrated in various clinical settings, detailed assessment of myocardial strain parameters would have provided incremental diagnostic and prognostic information as suggested in a study conducted by Sonaglioni et al. [30]

### Limitations

It is worth considering that our pilot study, has some limitations. First, patients were recruited from a tertiary center and only one patient had ejection fraction below 40%. This latter fact did not allow us to carry out a comparative analysis between patients with preserved and reduced ejection fractions categories.

Second, DR was classified as present or absent also due to the small sample size. This did not allow us to determine which degree of DR was more prevalent and more related with CAD.

Third, it was difficult obtaining adequate albuminuria samples for the determination of DRD according to the guidelines. We considered GFR reduction < 60 ml/min as the first stage of DRD. Finally, the results concerning the AUC for the level of triglycerides that are discriminatory for the presence of CAD were of moderate accuracy.

## Conclusion

In conclusion, this was a pilot study in an admixed population with low socioeconomic status and inadequate glycemic control, where the use of not invasive cardiovascular exams led to early detection of CAD in asymptomatic patients with T2D. The combination of CIMT and FRS score was more efficient in determining risk factors in asymptomatic patients with T2D. No echocardiogram parameter was able to detect the suggestive presence of CAD. Patients with DR and hypertriglyceridemia deserve further investigation for CAD. Further prospective studies with larger sample sizes are needed to confirm our results.

## Abbreviations

DM: Diabetes mellitus
CVD: Cardiovascular diseases
HF: Heart failure
T2DM: Type 2 diabetes mellitus
SMI: Silent myocardial ischemia
DR: Diabetic retinopathy
ECG: Electrocardiography
LV: Left ventricular
CAD: Coronary artery disease
FRS: Framingham risk score
VA: Vascular age
CIMT: Carotid intima-media thickness
CA: Chronological age
ADA: American Diabetes Association
LDLc: Low-density lipoproteins
DME: Diabetic macular edema
